# Efficacy and safety of freshly collected autologous adipose tissue for complex anal fistulas in non-IBD patients: a prospective cohort study

**DOI:** 10.1007/s10151-026-03297-6

**Published:** 2026-05-18

**Authors:** N. Sorensen, S. Buntzen, L. Pedersen, O. Thorlacius-Ussing

**Affiliations:** 1 Department of Gastrointestinal Surgery, Aalborg University Hospital, Hobrovej 18-22, 9000 Aalborg, Denmark; 2 Department of Clinical Medicine, Aalborg University Hospital, Aalborg, Denmark

**Keywords:** Adipose tissue/transplantation, Anal fistula/surgery, Autologous stem cells, Fecal incontinence/prevention, Prospective studies

## Abstract

**Background:**

Complex cryptoglandular anal fistulas present a treatment challenge, with many surgical options associated with recurrence, variable healing rates, and risk of incontinence. Freshly collected autologous adipose tissue (FCAAT) has been proposed as a minimally invasive, one-step alternative. This study aimed to assess clinical healing and clinical improvement and to evaluate the safety of the procedure.

**Methods:**

This prospective cohort study included 31 patients without inflammatory bowel disease (IBD) with complex single-tract cryptoglandular anal fistulas treated with FCAAT between May 2019 and December 2023 and followed until August 2024. The surgical procedure involved liposuction, processing of adipose tissue, closure of the internal opening, and local injection along the fistula tract in a one-step procedure. Primary outcomes were clinical healing and clinical improvement; secondary outcomes were adverse events.

**Results:**

The patient cohort presented with advanced disease. Median disease duration was 20 months, and one-third of patients had undergone previous attempts at surgical closure. Clinical healing was achieved in 23 patients (74%), with an additional four patients (13%) demonstrating clinical improvement. Five of 27 responders (19%) healed more than 3 months post-procedure.

Common adverse events included proctalgia in 8 patients (22%), donor site pain 5 (14%), and minor graft site hematomas in 4 (11%). One Clavien–Dindo IIIa event (graft site bleeding) was managed with a single suture; all other complications were minor and resolved conservatively.

**Conclusion:**

FCAAT is a safe and effective one-step treatment for complex anal fistulas in non-IBD patients, offering a high healing rate with predominantly minor complications. A delayed effect was observed in some patients.

## Introduction

Complex cryptoglandular anal fistulas are a persistent surgical challenge, causing significant morbidity and impairing quality of life. Current European Society of Coloproctology (ESCP) guidelines recommend a variety of sphincter-preserving procedures [1], yet healing and recurrence rates vary widely, and recurrence, postoperative complications, and risk of fecal incontinence remain common concerns [2, 3].

Autologous adipose tissue-derived stem cell therapy has demonstrated safety and efficacy in fistula treatment, but the process is costly, requires laboratory cell culture, and typically involves a two-stage procedure [4]. Freshly collected autologous adipose tissue (FCAAT), obtained via liposuction and injected during the same operation, has recently shown promising outcomes in both anovaginal and complex cryptoglandular fistulas in patients with and without inflammatory bowel disease [5–8]. FCAAT offers a potentially simpler, one-step, and less resource-intensive alternative to cultured stem cells.

This prospective cohort study aimed to assess the efficacy and safety of FCAAT for the treatment of complex cryptoglandular fistulas in non-IBD patients.

## Methods

### Study population

This prospective cohort study was conducted at the Department of Gastrointestinal Surgery, Aalborg University Hospital, Denmark. All consecutive non-IBD patients treated with FCAAT for complex single-tract anal fistula between May 2019 and December 2023 were included and followed until August 2024. Patients were either referred by their general practitioner or from emergency surgical departments within the region. Inclusion was based on clinical suitability for FCAAT treatment.

Fistulas were defined according to the Parks classification [9] and were considered complex if high intersphincteric or transsphincteric fistulas involving more than one-third of the internal or external sphincter, horseshoe fistulas, fistulas with secondary extensions, recurrent fistulas or anterior transsphincteric fistulas in women.

The Danish health system is publicly funded and universally accessible, with a small private sector that does not manage complicated fistulas. Electronic health records are thus considered comprehensive and up-to-date. No patients received FCAAT treatment at other institutions during the study or follow-up period. Disease onset was defined as the date of the first in-hospital record of a complex cryptoglandular fistula.

Baseline data were collected, including smoking status, diabetes, immune-modulating medicine (for concurrent rheumatic or dermatological conditions), fistula type according to the Parks classification, and history of previous fistula surgery [9]. Postoperative complications were graded according to the Clavien–Dindo classification [10].

### Inclusion and exclusion criteria

#### Inclusion criteria

Patients were eligible if they had a complex single-tract anal fistula with low inflammatory activity in the tract and an internal opening suitable for closure. Patients with secondary extensions, horseshoe fistulas, internal openings not suitable for tension-free closure, or severe inflammatory activity were initially treated with revision until the fistula was reduced to a single-tract fistula suitable for FCAAT.

#### Exclusion criteria

Patients were excluded if they had anovaginal fistulas, pouch-cutaneous fistulas or a confirmed diagnosis of IBD. Patients with suspected IBD were referred to the gastroenterology department for endoscopic and histological assessment. If diagnosed, they were excluded from the study.

#### Smoking cessation

As a result of the potential negative effect of active smoking on fistula healing, patients were required to abstain from smoking for ≥ 12 weeks before undergoing FCAAT. During the early phase of the study, three active smokers were treated; these cases are reported separately.

### Surgical procedure

#### Perioperative assessment

The FCAAT procedure was conducted as an outpatient surgical procedure. All patients underwent examination under general anesthesia by one of two colorectal surgeons (OTU or SB) and preoperative imaging with pelvic magnetic resonance imaging (MRI) and/or endoanal ultrasound to exclude secondary extensions or untreated abscesses. Loose seton treatment for at least 6–8 weeks was routinely applied to ensure low inflammatory activity before FCAAT.

#### Fat harvesting and preparation

FCAAT was obtained as a single-step procedure under general anesthesia. Fat was harvested from the flank region or lower abdomen after infiltration with an epinephrine–Ringer lactate solution (1:1,000,000) 15 min before harvesting. Manual liposuction was performed using a 3.5-mm cannula, followed by infiltration of 20 mL of ropivacaine (5 mg/mL) at the donor site. The harvested adipose tissue was centrifuged, the aqueous fraction removed, and the tissue homogenized by manually repeatedly transferring between two Luer-lock syringes.

#### Fistula preparation and injection

The fistula tract was thoroughly debrided using a curette and/or a fistula brush to remove granulation tissue. An absorbable suture was used to close the internal fistula opening. If the fistula tract was difficult to identify, a thin catheter was placed through the tract, and the absorbable suture was left untied until the end of the procedure. Two small incisions, approximately 2–3 mm in length, were made about 2 cm from the external fistula opening using a pointed surgical blade penetrating the skin. A 1.2-mm needle was inserted through the small incisions and used to inject a circumferential “donut” around the internal fistula opening. The procedure was performed under tactile and visual guidance to ensure adequate tissue swelling. The same needle was subsequently used to penetrate the fistula tract, with approximately 0.5 mL of FCAAT injected at each step, while gradually withdrawing the needle a few millimeters distally toward the external fistula opening. Perioperative antibiotics comprised dicloxacillin (2 g), metronidazole (1 g), and gentamycin (5 mg/kg).

#### Postoperative care

Postoperative antibiotics continued for 5 days with ciprofloxacin 500 mg twice daily. Patients received ascorbic acid (1000 mg daily) for 6 weeks post-procedure. Compression garments were advised for the donor site, and strenuous activities were avoided for 2 weeks. Analgesia consisted of ibuprofen and paracetamol as needed.

#### Follow-up

All patients were reviewed on postoperative day 1 to assess the donor site, followed by an outpatient visit 6–12 weeks after the procedure. Follow-ups were primarily conducted by the operating surgeon. If complete healing had not occurred, an additional visit was scheduled 8–12 weeks later. Patients demonstrating progressive healing were monitored with additional follow-ups until complete healing was achieved or a second procedure was deemed necessary.

Patients were considered clinically healed if they reported no discharge, had no visible external fistula opening, and no internal fistula opening was detected by digital rectal examination. Clinical improvement was defined as the presence of a small residual external opening without discharge or discomfort. These definitions were applied consistently throughout the study.

At each scheduled follow-up, patients were asked about postoperative adverse events. Reported events were documented in the electronic health record.

### Statistical analysis

All data were extracted from electronic health records and entered into a RedCap database [11]. Statistical analyses were performed using Stata (StataCorp. 2020. Stata Statistical Software: Release 16. College Station, TX: StataCorp LLC). Normality of continuous variables was assessed visually and with the Shapiro–Wilk test. Normally distributed data is presented as mean with 95% confidence intervals (95% CI), whereas non-normally distributed data are presented as median with interquartile range (IQR). Categorical variables are reported as counts and percentages. Comparisons between outcome groups (clinically healed, clinically improved, not healed) were performed using Fisher’s exact test for categorical variables and the Mann–Whitney* U* test for continuous variables. A *p* value < 0.05 was considered statistically significant. Adverse events were analyzed descriptively.

### Ethics

The study was registered with the Department of Research, Education and Innovation, Aalborg University Hospital (ID: K2023-016) and considered to be a quality assessment study that did not need approval by the regional research ethics committee. All patients provided informed consent for the procedure. All data were handled in compliance with the General Data Protection Regulation and local regulations governing patient confidentiality.

## Results

**Fig. 1:**
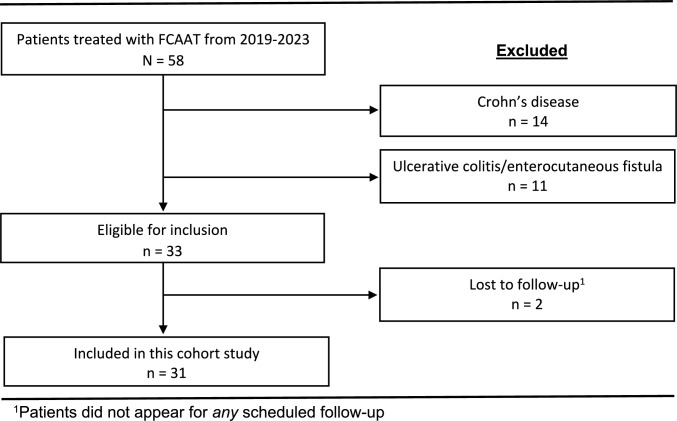
Flow diagram of included patients

Of the 58 patients treated with FCAAT, 25 were excluded because of inflammatory bowel disease (Crohn’s disease or ulcerative colitis) or anovaginal/pouch-vaginal fistulas. A further two patients were lost to follow-up (Fig. 1). The remaining 31 patients were included in the study. Baseline characteristics are summarized in Table 1. Patients were screened for alcohol or substance abuse but showed no signs of either. Two patients had preexisting diabetes. The median follow-up was 29 months (IQR 19–40 months) and the median injected volume was 32 mL.

**Table 1:** Patient and procedure characteristics (*n* = 31)

Age (years) (95%CI)	47.4 (42.8–51.9)
BodyMass Index (kg/m^2^ (95%CI)	28.1 (26.3–29.9)
Proportion of males (%)	60.6
Disease duration^1^ (months, IQR)	20.0 (14.0–43.5)
Patients with previous closure attempts, n (total procedures)	10 (13)
Smoking status n (%)	
Current smokers	3 (9.7)
Previous smokers	8 (25.8)
Never smokers	20 (64.5)
Type of perianal fistula (Park classification), n (%)	
High intersphincteric fistula	1 (3.2)
Transsphincteric fistula	30 (96.8)
FCAAT^2^ volume a first procedure mL, (95% CI)	32 (26.6–36.6)

No significant differences in age, gender, Body Mass Index or injected FCAAT were found between patients who healed, improved, or did not heal (all *p* > 0.05, data not shown)

^1^From first in-hospital record

^2^FCAAT: Freshly collected autologous adipose tissue

**Fig. 2:**
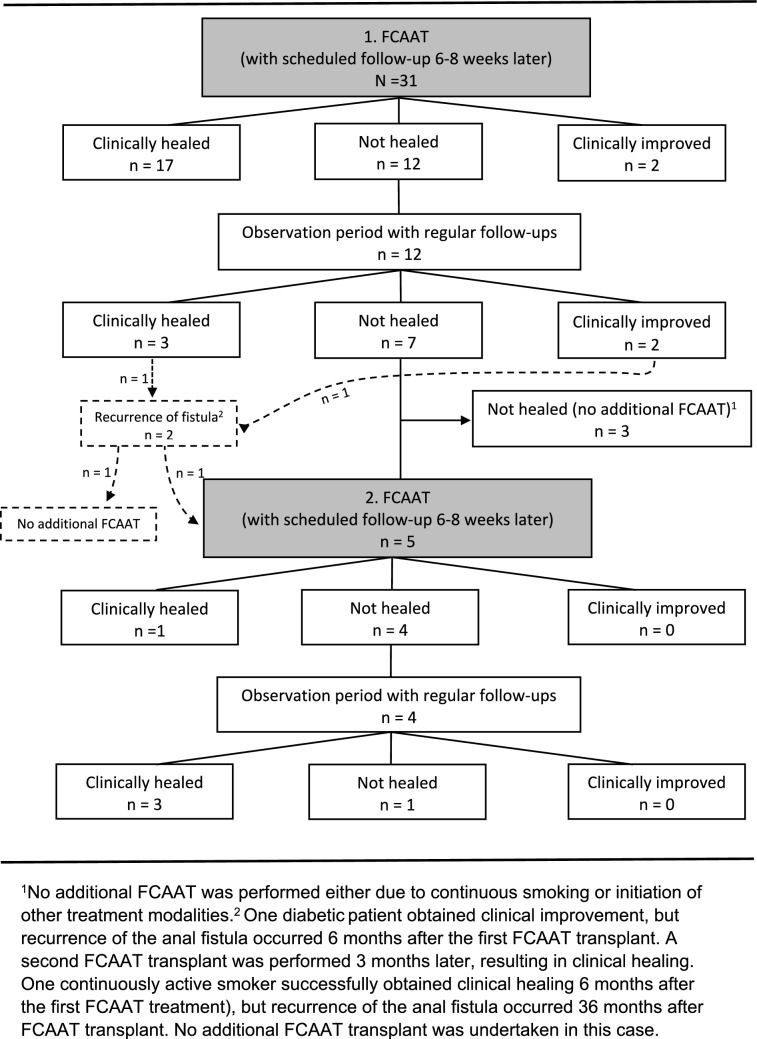
Flow diagram of patients through FCAAT transplants

Clinical healing was achieved in 23 patients (74%), and an additional 4 (13%) showed clinical improvement. The remaining 4 patients (13%) did not heal or improve. The median time to healing was 3.1 months (IQR 3.0–11.4 months) (Table 2). Of the 23 patients who healed, 21 did so after the first FCAAT procedure, including 17 at the first scheduled follow-up (6–8 weeks postoperatively). The patient flow is illustrated in Fig. 2.

**Table 2:** Results from FCAAT

Patient outcome^1^	
Clinically healed, n (%)	23 (74.2)
Clinically improved, n (%)	4 (12.9)
Not healed, n (%)	4 (12.9)
Number of treatments	
One,n (%)	26 (84.8)
Two,n (%)	5 (15.2)
Time to healing (in months), median (IQR)	3.1 (2.8–3.4)
Follow-up time (in months), median (IQR)	29.3 (19.1–40.3)

FCAAT, Freshly collected autologous adipose tissue

^1^Reported as best outcome during the study period. Two of the 23 clinically healed patients experienced relapse of their anal fistula. One was treated successfully with a second FCAAT. No additional FCAAT was initiated for the second patient due to active smoking status (please refer to the results section and Fig. 2)

Three active smokers were treated with FCAAT before the introduction of the ≥ 12 weeks preoperative smoking cessation requirement. Two of these patients achieved clinical healing or improvement. Among previous smokers and never smokers, 25 of 28 patients (90%) had a positive outcome (clinical healing or improvement) (Table 3).

**Table 3:** Healing rate according to smoking status

	Clinically healed or improved, n (%)	Not healed, n (%)
Smoker	2 (67%)	1 (33%)
Previous smoker	7 (88%)	1 (12%)
Never smoker	18 (90%)	2 (10%)

No significant differences in smoking status were found between patients who healed/improved or did not heal (*p* > 0.05, data not shown)

Two patients experienced recurrence of their anal fistula during follow-up. In the first case, the patient was followed for 27 months before clinical improvement was achieved; however, relapse with secretion occurred 6 months later. A second FCAAT was performed 46 months after the first procedure, resulting in clinical healing at the first follow-up (4 months postoperatively). In the second case, the patient was clinically healed at 6 months after FCAAT, but recurrence occurred at 36 months. This patient was an active smoker and continued smoking after the FCAAT procedure. No further FCAAT was initiated.

Ten patients (32% of the cohort) had undergone previous surgical attempts to close the fistula before FCAAT (Table 4). Three of these patients had been treated with multiple modalities, including combinations of Permacol plug, fistulectomy, LIFT, and advancement flap procedures.

**Table 4:** Surgical procedures before FCAAT treatment^1^

Permacol plug	5
Fistulectomy	5
LIFT^2^	2
Advancement flap	1

^1^FCAAT Freshly collected autologous adipose tissue. A total of 10 patients underwent 13 procedures

^2^Ligation of the intersphincteric fistula tract

### Postoperative complications

Postoperative complications are reported in Table 5. The most frequent adverse events were proctalgia in 8 patients (22%), donor site pain in 5 (14%), and minor hematoma at the graft site in 4 (11%). The most serious adverse event was a Clavien–Dindo grade IIIa bleeding from the graft site, which was treated with a single suture under local anesthesia. All other adverse events were minor (Clavien–Dindo grade I-II). “Other adverse events” included urinary retention in 1 patient, which resolved after a single catheterization, and nausea and vomiting in another patient for 24 h postoperatively, likely related to anesthesia. All hematomas were minor and resolved conservatively (Table 5).

**Table 5:** Adverse events (*n* = 36)

Proctalgia	8 (22)
Pain at graft site	5 (14)
Minor hematoma at graft	4 (11)
Other	2 (6)
Bleeding at FCAAT injection site	1 (3)
Flatus incontinence	1 (3)
Fecal incontinence	0 (0)
Abscess	0 (0)

A total of 31 patients undergoing 36 FCAAT procedures

*FCAAT* freshly collected autologous adipose tissue

## Discussion

FCAAT is a relatively new procedure, but several small cohort studies (*n* = 9–77 participants) have reported healing rates from 51% to 77% [5–7, 12]. In our study, the clinical healing rate was 74%, with an additional 13% of patients showing clinical improvement. These outcomes are in line with the published literature and are among the highest reported to date. However, direct comparisons are limited by substantial heterogeneity in inclusion criteria and definitions of healing across studies. In a cohort of patients with Crohn’s disease, Dige et al. demonstrated an overall response rate of 76% after FCAAT, while Norderval et al. found a 77% healing rate in a mixed population of patients with anovaginal fistulas [5, 7]. Guillaumes et al. showed a 66% healing rate, though only nine cases involved FCAAT [12]. The study most comparable to ours was conducted by Dalby et al., who reported a healing rate of 51% at 6 months in 77 patients, nearly half of whom received two FCAAT procedures [6]. The lower rate may be partly explained by their use of stringent healing criteria, which included mandatory postoperative pelvic MRI. In their series, an additional nine patients without MRI-confirmed healing had reduced discharge and anal discomfort, increasing the overall response rate to 60%. Although Dalby et al. demonstrated complete concordance between MRI and clinical healing, the value of MRI as a postoperative assessment tool remains uncertain, particularly given its time demands and limited availability. A complete correlation between pelvic MRI healing and clinical healing was demonstrated by Dalby et al., indicating that postoperative pelvic MRI is of limited value in cases of clinical healing at scheduled follow-up [6]. Direct comparisons between FCAAT and incubated stem cell therapy for complex anal fistulas in non-IBD patients are limited because of the paucity of comparable studies. In a series of 20 patients, MacIel Gutiérrez et al. reported complete closure in 69% and partial closure in 16% of patients after incubated stem cell therapy [13]. Herreros et al. found a complete healing rate of 57% (68 patients) 1 year after treatment; partial closure rates were not reported [14]. Overall, FCAAT seems to be at least comparable in efficacy to traditional stem cell therapy, with the added advantages of being performed as a one-step procedure and avoiding the need for complex cell incubation, thereby reducing associated costs. In addition to efficacy, the safety profile of FCAAT is a key consideration, and our findings align with the generally favorable complication rates reported in earlier studies.

Minor adverse events related to FCAAT were relatively common in our study, with the most frequent being proctalgia in 8 patients (22%), donor site pain in 5(14%), and minor hematomas at the graft site in 4 (11%). These rates are comparable to those reported by Naldini et al., who observed similar donor site pain and hematoma frequencies [15]. The relatively high incidence of reported proctalgia in our cohort may be partly attributable to the omission of local anesthesia in the fistula region, a deliberate choice intended to avoid potential interference with stem cell activity [16]. These minor complications were effectively managed with conservative measures. A single occurrence of flatus incontinence was reported at follow-up, but it is unknown whether the condition was related to FCAAT or a preexisting condition. As reported in the Results, the only major adverse event was a Clavien–Dindo grade IIIa graft site bleed, which was successfully managed with a single suture under local anesthesia.

Our baseline characteristics were generally comparable to those reported by Dalby et al. concerning mean age, fistula types, disease duration, and proportion with previous fistula closure attempt (around 30%), although our male proportion was higher. A noteworthy difference was the injected FCAAT volume, which averaged 32 mL in our study compared with mean or median volumes of 61–80 mL in previous reports [5–7]. The potential relationship between injected volume and treatment response remains unclear, and it is unknown whether lower-volume FCAAT injections could result in higher healing rates. While higher volumes might theoretically deliver a greater number of regenerative cells, lower volumes could facilitate more precise placement and reduce tissue pressure at the graft site, potentially improving graft integration and survival.

An interesting observation in our cohort was the potential for late healing after FCAAT. Of the 12 patients not healed at the first scheduled follow-up (at 6–8 weeks), 5 achieved clinical healing or improvement without further treatment (Fig. 2). The latest case of clinical healing occurred almost 27 months after the initial procedure. This delayed response should be considered when determining the timing for a potential second FCAAT. In contrast, Dige et al. offered a second FCAAT treatment as early as 6 weeks after the first FCAAT, if unsuccessful [7], which may have precluded identification of later responses. It is possible that a second, early FCAAT with curettage could even be detrimental to healing, which might explain the higher healing rates observed in our study. Other factors that may influence treatment response include patient-related variables, such as smoking status, which has been proposed as a negative prognostic factor in both FCAAT and other fistula treatments.

Active smoking remains a debated prognostic factor in fistula surgery and in regenerative approaches such as FCAAT. In our study, three active smokers were treated before the introduction of the ≥ 12-week preoperative smoking cessation requirement. Two achieved clinical healing (albeit with one late recurrence of the fistula), while one did not respond to treatment. Dalby et al. reported a significantly lower healing rate among seven included smokers (0%) compared with never smokers (*p* < 0.05) [6] and a recent meta-analysis of stem cell-based treatments for anal fistulas found a significantly poorer healing rate in smokers (*p* < 0.001) [17]. In general, the impact of smoking on healing after traditional anal fistula surgery remains debated. Schwandner et al. conducted a prospective, multicenter study on transsphincteric anorectal fistula treated with plug closure, reporting that smoking significantly decreases the rate of healing. In contrast, a large meta-analysis by Mei et al. found that “high quality evidence demonstrated no significant association with smoking” when assessing risk factors for recurrence after anal fistula surgery [18, 19]. As detailed in the Methods section, smoking cessation for at least 12 weeks prior to FCAAT was mandated during the study period because of the potential negative impact on healing. This criterion was introduced after the early treatment phase, in which three active smokers were included; their outcomes are reported separately. Given the heterogeneity of published data on smoking and anal fistula outcomes [6, 17–19], adherence to this preoperative requirement should be considered when interpreting our high overall healing rate. Future studies could formally evaluate the effect of structured preoperative smoking cessation on FCAAT outcomes.

Care should be taken when extrapolating our 88–90% “success rate” for treating complex anal fistula in non-smokers with FCAAT. Our definition of “clinical improvement” involves both subjective judgment by the examining surgeon and the patient’s reported postoperative symptoms. Moreover, the definition of “complex anal fistula” varies between studies, and differences in patient populations might lead to inconsistent results. The use of structured MRI and endoanal ultrasound reporting templates for anal fistulas (SMART) [20] was not standard practice at the beginning of the study period, and data derived from such templates are therefore not reported. In general, few research papers report data such as the exact height of the internal fistula opening etc., but consistent reporting of SMART data might be beneficial to identify the best surgical approach to each patient and be advantageous when comparing anal fistula complexity across studies. The reported healing rates of more traditional surgical approaches—such as fistulectomy with or without primary sphincter reconstruction, ligation of the intersphincteric tract, or advancement flaps—vary widely, ranging from 24% to 100%, depending on the surgical technique, patient population, and duration of follow-up [3]. Notably, a recent single-center study reported a high healing rate of 84% (comparable to our study) in the treatment of complex non-IBD anal fistulas using core-out fistulectomy, without any associated fecal incontinence; however, the recurrence rate was relatively high at 23% [21].

In our cohort, the median disease duration was 20 months, and one-third of patients had undergone previous surgery, indicating a level of disease complexity comparable to that reported in other similar study populations [6, 21]. Naldini et al. found a healing rate of 83% among patients treated with micro-fragmented adipose tissue as first-line treatment, but only 57% in those with prior surgery [15]. Similarly, Mei et al. found that prior surgical treatment increased the risk of recurrence after anal fistula surgery [19]. A similar association may apply to FCAAT, although it was not observed in our study, possibly as a result of the limited cohort size. Larger, controlled trials with standardized outcome measures are warranted to confirm these results and optimize patient selection and treatment protocols.

## Conclusion

FCAAT is a safe, minimally invasive, one-step treatment for complex cryptoglandular anal fistulas in non-IBD patients, demonstrating high healing rates and a low risk of adverse events. Its technical simplicity, favorable safety profile, and potential for delayed healing suggest that FCAAT may represent a promising alternative to incubated stem cell therapies and established surgical techniques. Larger, controlled studies are warranted to confirm these findings and to refine patient selection criteria.

## Data Availability

No datasets were generated or analysed during the current study.
